# Benefits of annual chemotherapeutic control of schistosomiasis on the development of protective immunity

**DOI:** 10.1186/s12879-019-3811-z

**Published:** 2019-03-04

**Authors:** Tawanda J. Chisango, Bongiwe Ndlovu, Arthur Vengesai, Agness Farai Nhidza, Edson P. Sibanda, Danai Zhou, Francisca Mutapi, Takafira Mduluza

**Affiliations:** 1 School of Laboratory Medicine and Medical Sciences, College of Health Sciences, University of KwaZulu-Natal, Durban, South Africa; 2 Biochemistry Department, University of Zimbabwe, 630 Churchill Ave, Mount Pleasant, Harare, Zimbabwe; 3 Scientific and Industrial Research and Development Centre, 1574 Alpes Road, Box, Harare, 6640 Zimbabwe; 4 Medical Laboratory Sciences, College of Health Sciences, University of Zimbabwe, Harare, Zimbabwe; 5 Institute of Immunology & Infection Research, University of Edinburgh, Ashworth Laboratories, King’s Buildings, Charlotte Auerbach Road, Edinburgh, EH9 3FL UK

**Keywords:** Praziquantel, Schistosomiasis, MDA, Treatment, Antibodies, Immunity

## Abstract

**Background:**

Schistosomiasis is a devastating parasitic disease. The mainstay of schistosomiasis control is by praziquantel treatment. The study aimed to determine benefits of annual chemotherapy of schistosomiasis on development of protective immunity in school children in a selected endemic rural area in Zimbabwe.

**Methods:**

Urine specimens from 212 school children (7–13 years) were collected and examined to determine prevalence, intensity and reinfection of *S.haematobium* at baseline, 6 weeks and 2 years following annual rounds of praziquantel treatment*.* Blood samples from the participants were assayed for total and *S. haematobium* (Sh13)-specific antibodies before and 2 years after annual rounds of treatment.

**Results:**

Annual treatment reduced the prevalence of *S. haematobium* infection (*p* < 0.05) from 23.1% at baseline to 0.47% after 2 years. Overall cure rate was 97.8%. Intensity of infection declined (p < 0.05) from 15.9 eggs/10 ml urine at baseline to 2 eggs/10 ml urine. After two years, overall rate of reinfection was 0.96%. At baseline, total IgG4 was higher in *S. haematobium*-infected children (*p* = 0.042) ,while all other immunoglobulins were within normal ranges. There was an increase in total IgG2 (*p* = 0.044) levels and a decrease in total IgG4 (*p* = 0.031) levels 2 years post-treatment; and no significant changes in other total immunoglobulins. *Schistosoma*-infected children at baseline showed an increase in anti-*Sh*13 IgG1 (*p* = 0.005) and a decrease in *Sh*13 IgG4 levels (*p* = 0.012) following treatment.

**Conclusion:**

Annual praziquantel treatment delivered to school children over 2 years significantly reduce prevalence, intensity of infection and reinfection of *S. haematobium* infection. Treatment was also observed to cause a reduction in schistosome-specific blocking IgG4 and an increase in *Schistosoma*-specific protecting IgG1.

**Electronic supplementary material:**

The online version of this article (10.1186/s12879-019-3811-z) contains supplementary material, which is available to authorized users.

## Background

Schistosomiasis is a devastating neglected tropical disease (NTD) that begins when infective cercariae from freshwater snails pierce the skin as a result of exposure to infested water [1]. Such a scenario is typical in resource limited settings with poor sewage disposal and inadequate supply of clean water. It is estimated that over 250 million people are infected with schistosomiasis, worldwide. Approximately 93% of the infected people reside in sub-Saharan Africa [2], where school children carry the heaviest burden and account for the highest prevalence and heavy intensity of schistosome infections [3]. Children are mainly infected due to their higher rates of water contact activities in open water sources and immature immunological status [4, 5]. The infected children usually suffer from haematuria, dysurea, several nutritional deficiencies and anaemia that affect growth, decreased physical performance and impaired memory and cognition when the schistosome infection intensity becomes heavy [6, 7].

The Ministry of Health and Child Care in Zimbabwe included schistosomiasis in the 2009–2013 National Health Strategy in 2009 underlining the importance and the urgent need to control the disease. A nationwide schistosomiasis survey was conducted in Zimbabwe and the overall prevalence of *S. haematobium* was reported as 18.0% while that of *S. mansoni* was 7.2%. Manicaland region, where this study was conducted has a moderate burden of the parasitic disease at 23.8% prevalence of schistosomiasis [7, 8].

Chemotherapeutic control using praziquantel is aimed particularly at school age children living in schistosomiasis endemic areas. The treatment and control measure for schistosomiasis has been recommended by the World Health Organization (WHO) [9] as an interim available control strategy, since no vaccines are as yet available. Praziquantel is a widely used drug for treating schistoisomiasis and very few cases of treatment failures have been reported. Generally praziquantel is easy to administer, safe, well tolerated, cheap, can reverse schistosome-related morbidity and is highly effective against the five schistosome species that infect humans [10, 11]. Mass drug administration using the anti-helminthic drug praziquntel has been the major focus of recent control efforts [12], with the principal aim of reducing morbidity and clearing sources of recontaminating the environment. Scaling up of mass drug administration has been proposed in the WHO’s strategic plan of 2012 as a way of managing schistosomiasis morbidity by 2020 [13]. Schools have been targeted for mass treatments because of the increased benefits of reducing infection burdens in children compared to adults and the simplicity of providing treatment [14]. Many studies have shown that praziquantel drastically reduces morbidity and transmission of schistosomiasis especially in low-to-moderate transmission areas where the risk of reinfection is generally low [15–17], despite a few reports of treatment failures [18, 19].

Although praziquantel is efficient in removing active infection, it does not have an effect on the developing immature worms. As a result, reinfections have been reported and still remains as a challenge in communities where it is unavoidable to get in contact with water infested with snails carrying the *S. haematobium* cercariae [20]. In high transmission endemic areas, the first treatment using praziquantel must be divided over weeks to also target the immature worms and repeatedly administered over rounds of annual treatment for a period in order to maintain low reinfection levels [21], however the long-term effects on infection dynamics and immune status are not yet fully understood. In our current study, repeated annual rounds of praziquantel were administered to children attending Bandanyenje primary school. Although the overall prevalence and distribution of schistosomiasis have been reported in the province, less emphasis has been given to the effect of repeated rounds of praziquantel and the subsequent prevalence, intensity of infection and re-infection rates in Bandanyenje. Information on the efficacy of praziquantel and the infection rates may help in evaluating policies and strategies that guide schistosomiasis control activities in the district. Thus, we assessed the effect of annual treatment with praziquantel over 2 years on the prevalence and reinfection rates in school children attending Bandanyenje primary school in Manicaland province of Zimbabwe. The intensity of *Schistosoma* infection, using the major symptom, haematuria, as an indicator of morbidity, was also evaluated.

Several studies have reported the chemotherapy-induced changes in responses to schistosome infection [13, 22, 23]. Frequent treatments are thought to enhance the development of protective immune responses. Studies of the effects of chemotherapy on other helminths indicate that the drug treatment can facilitate the development of resistance to re-infection through enhanced production of protective antibodies. Key information on the humoral immune status of individuals can be determined routinely by measuring total immunoglobulin levels. Several studies have realized the association between total immunoglobulins in chronic diseases associated with inflammation such as diabetes [24, 25]. Very little has been done on the association between the levels of total immunoglobulins and schistosomiasis, which is also a chronic disease that is associated with inflammation. Indeed treatment of schistosomiasis has shown to be associated with subsequent changes in humoral and cellular immune responses although focus has been mainly on *Schistosoma*- specific antibodies [22]. Antibodies such as IgE, IgM and IgG1 have been shown to be associated with resistance against schistosomiasis (4, 18–20), while IgG2 and IgG3 have been shown to kill schistosomula in the presence of activated eosinophils (21). IgG4 has been suggested as a modulator for the anaphylactic responses associated with IgE (22, 23) while IgA responses have been shown to be associated with reduced parasite fecundity (24). Analysis of such changes in the context of both total and schistosme-specific antibodies will assist in interpreting the differences that occur in children living in endemic areas as well as improving current understanding of the development of acquired immunity to schistosome infections and contributing towards vaccine development. In order to establish the relationships between total serum immunoglobulins and *S.haematobium-*specific antibodies and to better understand antibody turnover after praziquantel treatment we quantitatively measured the total immunoglobulins and *Schistosoma*-specific antibody profiles in school children before and after two annual rounds of treatment using praziqunatel.

## Methods

### Study area and population

The study was carried out in Bandanyenje Primary School located in the Manicaland Province in Zimbabwe. The school is approximately 217 Km South-East of Harare with latitude and longitude of 7°1′N 38°35′E. Villagers living in Bandanyenje community depend on perennial rivers as their water source thus indicating an increased likely exposure of the majority of their population to infection. The study population comprised of 212 (105 boys and 107 girls) aged between 7 and 13 years, who were permanent residents of the area.

### Parasitology and blood sampling

A school-based longitudinal intervention study was conducted and involved examination and treatment of the study population at baseline, 6 weeks and at 2 years follow up surveys. Stool and urine samples were collected over three consecutive days at baseline and at follow up, for each examination time-point. The urine samples were processed for urinary schistosomiasis using the filtration technique following Mott et al., 1982 [26] method. The stool samples were processed following the Kato Katz technique and Formol-ether concentration method as modified by Peters et al., (1980) [27]. A sample with the number of eggs greater than zero in 10 ml of urine was classified as being *S. haematobium*-positive. Those found to be infected with *S. mansoni* were excluded from the study; however the sampling site was observed to have very low *S. mansoni* transmission. The prevalence was defined as the number of infected children with *S. haematobium* over the total number screened. Intensity of *S.haematobium* infection was expressed as number of eggs per 10 ml (ep10ml) of urine. Infection intensities were classified into three categories: (1) light infections (< 10 ep10ml), (2) moderate infections (10 ep10ml < x < 50ep10ml and (3) heavy infections (≥ 50 ep10ml). Blood samples were obtained from the children, the serum separated and used to determine total immunoglobulins and antibody profiles against *S. haematobium* 13 (Sh13).

### Determination of plasma Total antibody profiles

About 50 μl 1x beads were added to a 96 well micro titer plate and washed 2x with the Bio-Rad wash buffer, (Cat #, 10,014,939). The standards, samples and controls were diluted (Bio-Rad Antibody diluent, (Cat #, 35,002,989) and 50 μl added to the wells and incubated in the dark at room temperature with shaking at 850 rpm for 1 h. After incubating the beads, samples, standards, blank and controls; the plates were washed three times with 100 μl wash buffer. 1x detection antibodies were added to the assay plate and incubated in the dark for 30 min with shaking at 850 rpm. The plate was washed 3x and Streptavidin – PE (Cat #, L9703897) was added. The assay plate was incubated for 10 min at room temperature with shaking at 850 rpm. The plate was washed 3x and the beads (Bio-Rad Cat #, 171–304,040) were re-suspended for plate reading. The Bio-plex manager software was used running the assay, data acquisition and analysis.

### Determination of *S. haematobium* (Sh13) specific antibodies

The recombinant Sh13 protein was used for the immuno-detection of specific antibodies patterns among 147 school children aged 7–13 years old and exposed to *S. haematobium*. The anti-schistosome recombinant vaccine candidate (Sh13) has been postulated to prevent worms from pairing thereby preventing them from laying eggs. The Sh13 cDNA sequence has been described and deposited in the Genbank database at the NCBI under the bankit no DQ**709821.** An indirect ELISA was optimized and used to quantify the amount of antibodies IgG1, IgG4 and IgE produced directly against the Sh13 antigen. Both pre- and post –treatment samples were analyzed on the same plate. Briefly, the plates were coated with Sh13 antigen at 5 ng/ml over night at 4 °C. Diluted plasma was added at 1:10 for IgG4, 1:50 for IgG1 and IgE, dilutions. Monoclonal anti-human IgG conjugated to peroxidase was used to detect the presence of anti-Sh13 IgG1, IgG4 and IgE antibodies. Six microwells were reserved for positive, negative controls and background blank containing MBP in duplicate, that was used in the preparation of the Sh13 antigen. This was due to the Sh13 suspension contain MBP. About 100 μl of the OPD substrate was added to each well containing horse reddish peroxidase enzyme and incubated for 30 min in the dark. The enzyme reaction was stopped with sulphuric acid and the absorbance was read at 450 nm with 630 as reference wavelength.

### Haematuria

As a measure for schistosome-induced pathology, haematuria was determined in urine samples collected at baseline and 2 years following treatment with 40 mg/kg praziquantel. A Combur-Test (Roche Diagnostics GmbH, Mannheim, Germany) reagent strip was used to detect the presence of blood in the urine.

### Treatment

Treatment was carried out by qualified health teams of trained nurses and medical officers as part of the MDA treatment campaign. All school children were treated with a single dose of 40 mg/kg praziquantel at baseline and thereafter without considering the infection status after 1 and 2 years.

### Ethical considerations

Blood and urine specimens were obtained from willing children who agreed to participate in the study following the signing of informed consent forms by their parents and guardians. Ethical approval for the study was obtained from the Medical Research Council of Zimbabwe (MCRZ) (Approvals MRCZ/A/1710 and MRCZ/A/1958). In addition, Provincial Medical and Education Directors, councillors and village head-men granted permission for the study.

### Data analysis

Data was entered into the computer using Microsoft Excel spreadsheet and exported to SPSS for windows version 16.0 (SPSS Inc., Chicago, Illinois, USA). The Pearson chi-square test was used to compare the differences in the prevalence of infections and arithmetic egg counts as well as to assess the association with age and gender. Student t-test was used to compare differences in the prevalence and intensity of infection (eggs/10 ml urine) before and after treatment. A value of *p* < 0.05 was considered as statistically significant. To determine if there was a significant difference between pre-and post-treatment of the total immunoglobulin as well as the specific antibody profiles a comparison of means using a paired t-test was conducted. The hypothesis that there were no differences between pre-treatment and 2 years post treatment antibody profiles was tested. *P* value set at *p* < 0.05.

## Results

### Baseline prevalence, intensity and reinfection

Of the 233 school children from Bandanyenje primary school who volunteered and provided urine and stool samples, *S. mansoni* was diagnosed in (9%) 21/233 and soil transmitted helminths in 0.4% (1/233) and these children were not included in the study. The remaining 212 (105 boys and 107 girls) aged between 7 and 13 were recruited in the study and were successfully traced and re-examined at both follow-ups with complete sets of longitudinal parasitological data on *S. haematobium* infection. The overall pre-treatment prevalence of *S. haematobium* infection was 49 (23.1%) with 20 (19%) boys and 29 (27.2%) girls as determined using the urine filtration technique. The age group of 10–13 years as well as the girls had the highest prevalence 25.6 and 27.1%, respectively. However the differences in infection status between age groups (χ^2^ = 0.158, *P* = 0.691) and boys and girls (χ^2^ = 1.891, *p* = 0.169) were not significant (Table 1). The majority of infected children in the both age groups had light infections with more heavy infections being observed in the 7–9 age (10.5%) group than the 6.7% in the 10–13 age group. However the intensity of infection had no age group (χ^2^ = 2.594, *p* = 0.273) or sex-related pattern (χ^2^ = 0.297, *p* = 0.862). The proportion of boys (10%) that had a heavy infection was greater than girls (6.9%) though not statistically significant (Table 1).

**Table 1:** Baseline prevalence and intensity of *S. haematobium* infection

Parameter	*S. haematobium* infected	*S. haematobium* uninfected	χ^2^	*P* value	Light Infection	Moderate Infection	Heavy Infection	χ^2^	*P* value
Sex			1.891	0.169				0.297	0.862
Boys	20 (19%)	85 (81%)			11 (55%)	7 (35%)	2 (10%)		
Girls	29 (27.1%)	78 (72.9%)			20 (69%)	7 (24.1%)	2 (6.9%)		
Age Group			0.158	0.691				2.594	0.273
7–9	19 (20%)	76 (80%)			13 (68.4)	4 (21.1%)	2 (10.5%)		
10–13	30 (25.6%)	87 (74.4%)			18 (60%)	10 (33.3%)	2 (6.7%)		

There was an overall decline in *S. haematobium* prevalence from 23.1% at baseline to 0.47% % at 6 weeks post-treatment and, 0.47% 2 years post-treatment, an overall 87.1% reduction over 2 years (*p* < 0.05). A total of 211 (99.5%) children were egg negative after treatment. Boys had a higher baseline *S. haematobium* prevalence, although it was not statistically significant. The *S. haematobium* prevalence significantly decreased to 0.47% (p < 0.05%) following 2 years of 3 annual rounds of praziquantel treatment. The overall Cure Rate (CR) was 97.8% and egg infection intensity was 15.9 egg/10 ml urine at baseline and reduced to 2 egg/10 ml urine 2 years post-treatment (Table 2). The study of reinfection involved the 211 children who were cured 6 weeks after the first praziquantel treatment. Of these *S. haematobium* negative, only 1 boy (0.96%) contracted the infection 2 years after the 6 week treatment (Table 3). Apparently this was a boy who had a heavy infection (60 eggs/10 ml) at baseline.

**Table 2:** Prevalence, cure rate and egg intensity at baseline, 6 weeks and 2 years following annual treatment with praziquantel

Variable	Baseline	6 weeks post treatment	2 years post treatment
Prevalence Cure rate	49 (23.1%)	1 (0.47%) 98%	1 (0.47%) 97.8%
Boys Cure rate	20 (19%)	1 (0.47%) 95%	1 (0.47%) 94.7%
Girls Cure rate	29 (27.1%)	0 100%	0 100%
7–9 Cure rate	19 (20%)	1 (0.47%) 94.7%	1 (0.47%) 94.4%
10–13 Cure rate	30 (25.6%)	0 100%	0 100%
Eggs per 10 ml	15.9	3	2

**Table 3:** Re-infection cases of *S. haematobium* post-praziquantel treatment

	*S. haematobium* infected	*S. haematobium* uninfected	Reinfection Cases
6 weeks			
Boys	1 (0.95%)	104 (99.05%)	–
Girls	0 (0%)	107 (100%)	–
2 years			
Boys	1 (0.95%)	103 (99.03%)	1 (0.96%)
Girls	0 (0%)	107 (100%)	0 (0%)

### Haematuria

Haematuria was detected in 32 (15.1%) of the study participants of which 23 (71.9%) were *S. haematobium* infected. The presence of *S. haematobium* eggs showed statistically significant association (χ^2^ = 20.38, *p* < 0.05) with haematuria whilst there was no association between sex and haematuria (χ^2^ = 0.08, *p* = 0.929). At 2 years post treatment, the extent of haematuria decreased from 100 to 0%.

### Total and *S. haematobium* specific antibodies

**Fig. 1:**
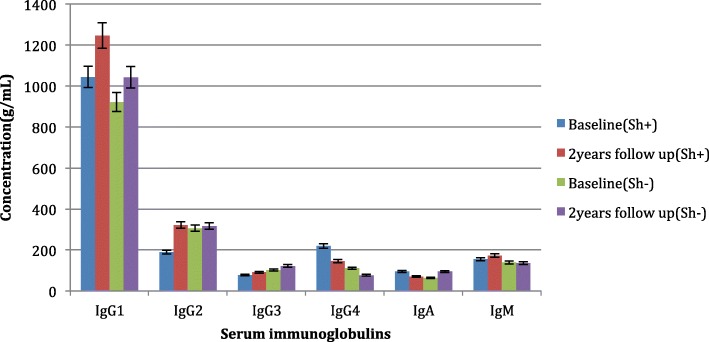
Concentration of total antibodies at baseline and 2 years post annual praziquantel treatment

**Fig. 2:**
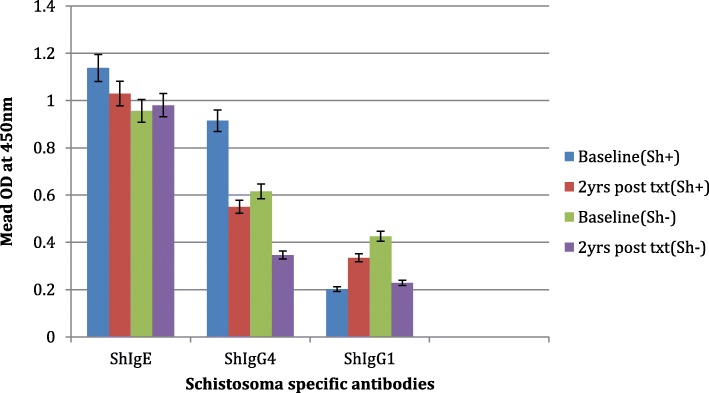
Mean OD values of *S. haematobium* specific antibodies at baseline and 2 years post annual praziquantel treatment

Baseline levels of total IgG4 were significantly higher in *S. haematobium* infected compared to *S. haematobium* negative children (Fig. 1). At 2 years after treatment, serum IgG2 increased and serum IgG4 tended to decreased post treatment with praziquantel (*p* = 0·044 and 0·031, respectively). There were no significant changes in total plasma IgG1, IgG3, IgM and IgA. Levels of *S. haematobium* IgG4 were significantly higher in *S. haematobium* infected when compared to *S. haematobium* negative (*p* < 0.05). Following chemotherapy, significant decreases of *S. haematobium* IgG4 were observed. Specific *S. haematobium*-IgE levels before treatment were higher than the *S. haematobium* IgE levels though no significant changes were noted before and after treatment in both *S. haematobium* uninfected and infected children (Fig. 2). The protective *S. haematobium* IgG1 increased significantly in *S. haematobium* infected children following treatment.

## Discussion

At baseline survey, the prevalence of *S. haematobium* infection in the study area was observed to be low at 23.1% compared to other areas in Zimbabwe [8]. However, the prevalence was within the range of the average prevalence within the province (Manicaland, 23.8%) as determined by a nationwide survey that was conducted by Midzi et al., (2014) [8]. The low prevalence of *S. haematobium* infection reported in this study could reflect the on-going control efforts in the area. Lately, constant awareness campaigns advocated during the annual mass drug administration exercise compounded by improved health education in rural areas have been part of efforts by the Ministry of Health to control schistosomiasis which had been neglected for a long time. This could have contributed in decreased contact with infested waters by children in the area.

Infection intensity is a better indicator of morbidity associated with schistosomiasis than prevalence as it indirectly reflects the number of worms infecting the individual and it is also a more reliable marker of treatment success defined as the removal of egg-laying worms [28–32]. We observed a significant decline in prevalence after the first dose of treatment at 6 weeks exhibiting a satisfactory efficacy with praziquantel. In this study, our results demonstrated a significant impact of praziquantel in treatment of *S. haematobium* with cure rates of 97.8% (*p* < 0.05%) following 2 years of repeated annual rounds of praziquantel treatment. This is comparable to findings of similar studies that have reported a higher efficacy of praziquantel when administered as two or three treatments spaced at certain time intervals [14, 33].

Total prevalence of re-infection 2 years post-treatment was 1%. It has been hypothesized that, after infections and repeated rounds of praziquantel chemotherapy, humans slowly acquire protective immunity to *S. haematobium* leading to partial resistance to re-infection [34]. Treatment with praziquantel boosts immunoglobulin E (IgE) antibodies against adult worms which are associated with resistance to re-infection [17]. In contrast to our findings, other studies observed a rapid and high re-infection rate a few weeks following treatment especially in high transmission areas [35–37]. Presence of haematuria before treatment was correlated with the presence of *S.haematobium* eggs in urine. In response to treatment with single dose of praziquantel, haematuria fell from 100 to 0% at 2 years post- treatment (Table 4).

**Table 4:** Occurrence of haematuria at baseline and 2 years after praziquantel treatment

	Before Treatment		2 years Post treatment	
	*S. haematobium* Infected	*S. haematobium* Uninfected	*S. haematobium* Infected	*S. haematobium* Uninfected
Boys	9 (45%)	5 (5.9%)	0	0
Girls	14 (48.3%)	4 (5.1%)	0	0
7–9	8 (42.1%)	3 (3.9%)	0	0
10–13	15 (50%)	6 (6.9%)	0	0

There is mounting evidence that anti-helminthic treatment using praziquantel not only transiently reduces infection, but also has longer term benefits in terms of morbidity control and the development of parasite-specific immune responses associated with resistance to re-infection [18, 38]. It is widely accepted that the changes in *Schistosoma*-specific immune response occur following treatment of schistosomiasis with praziquantel [7, 38, 39]. Praziquantel penetrates the tegument of worm tissues and rapidly moves through damaging the tegument and causing paralysis of the worm [40]. The increase in antigens released from dying worms as a result of praziquantel induced-tegument damage is believed to trigger this change in both the cellular and humoral immune responses [41].

In this study following chemotherapy there were changes in both the total and *S. haematobium*-specific antibodies. There was a significant increase in total IgG2, total and *S. haematobium-*specific IgG1 and a significant decline in total and *S. haematobium*-specific IgG4 while there were no significant changes in all the other antibodies. Antibody responses against Sh13 were predominantly IgG3 and IgG1 isotypes indicating establishing a protective immunity compared to responses, which were predominantly IgG4 in the reinfected. Studies done in individuals with chronic schistosomiasis have revealed elevated levels of total immunoglobulins [42]. We observed an increase in both the total and specific IgG1 which was maintained over the 2 years, which suggest that IgG1 could have been stimulated by antigens produced from the worms after chemotherapy. Khalife et al.*,* (1989) [45] demonstrated the eosinophil-dependent killing of schistosomula as a result of IgG1. This could explain the significant increase of IgG1 following treatment with praziquantel and the significant clearance of infection at 6 weeks and thereafter 2 years later. The release of sub-surface antigens and a decline in egg counts following treatment result in stimulation of IgG1 production. A similar increase in specific IgG1 following chemotherapy was noted by Mutapi et al (1998) [48]. They observed an increase in IgG1 following treatment with praziquantel, which they attributed to a switch from IgA specific antibodies to IgG1 response that occurs in children. The potential mechanism for this switch is not fully understood, but it is believed that changes in cytokine levels in response to antigen release from damaged parasites may cause this isotype switch from IgA to IgG1 [23]. The switch occurs naturally as worms die but takes a long time as a result of life span of schistosomes. The switch is accelerated by the praziquantel treatment which actually causes partial protection to reinfection as seen by the 1% reinfection rate 2 years post treatment noted in this study. The changing relationship between infection intensity and anti-Sh13 IgG3 levels post treatment is consistent with the profile of a protective immune response predicted from theoretical work and with protective immunity developing with cumulative experience of parasite antigens after rounds of MDA.

Most studies have demonstrated the protective role played by IgE, which surprisingly in this study showed no significant change in both the infected and non-infected group at baseline. Following treatment there were no significant changes (*p* < 0.05) in IgE in both *S. haematobium* infected and non infected (Fig. 1), though the levels remained higher than blocking IgG4 levels that decreased significantly following chemotherapy. Similar trends though in *S. mansoni* were observed by Walter et al.*,* (2006) [49]. They demonstrated that following treatment, adult worm-specific IgG4 levels decreased, while worm-specific IgE are maintained at pre-treatment levels or increases even in children, who more readily become reinfected, treatment is less likely to increase the IgE/IgG4 ratio. There is need therefore to carry out further studies especially taking into account cytokine levels which are key to facilitating the production of both the total and *S. haematobium*-specific antibodies.

In our study *S. haematobium* uninfected children had significantly lower levels (*p* < 0.05) of the blocking antibody IgG4 at baseline than the infected children, this was observed for both total and *S. haematobium*-specific IgG4 (Figs. 1 and 2). Blocking antibodies like IgG4 which have been observed to develop early in life have been shown to predispose children to infection. Studies on *Schistosoma*-infected populations have reported that anti-*Schistosoma* IgG4 levels in infected children are associated with higher parasite burdens and parasite susceptibility [43, 44]. IgG4 is known to interfere with IgE-induced mast cell and eosinophil degranulation through preventing the binding of IgE to the effector cells thereby preventing killing of schistosomula [45], IgG4 will also block IgG1 and IgG3 mediated killing of schistosomula by human eosinophils in vitro [34]. Elevated IgG4 levels observed in *S. haematobium* infection compared to uninfected children increase predisposition to infection. Following treatment there was a significant decrease (*p* < 0.05) in IgG4 highlighting the importance of praziquantel in lowering IgG4 which then results in a significant decline in infection at 6 weeks and 2 year post treatment. There is need therefore to carry out further studies especially taking into account cytokine levels which are key to facilitating the production of both the total and *S. haematobium*-specific antibodies.

## Conclusion

This study confirms findings from previous work by other research groups that praziquantel treatment reduces *S. haematobium* egg burden and alters immune responses. There was a significant reduction in prevalence, intensity of infection and reinfection. Since the study area is a moderate zone of transmission without any specific past control program, treatment with praziquantel once every 2 years may keep the infection at low level of transmission. Two years of annual praziquantel treatment significant changes occured in total and *Schistosoma*-specific IgG1 as well as total and *Schistosoma*-specific IgG4 changes. These findings suggest long term health consequences of praziquntel effects in changing the overall protective immunity in the school children and benefits of the repeated mass drug administration.

## Additional file

Additional file 1: 1. Parasitology egg counts pre and post treatment (Sh and Sm) and STH. 2. Sh13 Antibodies Optical Density measurements. 3. Total Antibodies Concentrations. 4. Acute Phase Proteins Concentratons. 5. Cytokine Concentrations. (XLSX 27 kb)

## Abbreviations

CR: Cure rate
MDA: Mass drug administration
NTD: Neglected tropical diseases
PZQ: Praziquantel
Sh13: *S.haematobium* 13
WHO: World Health Organization
